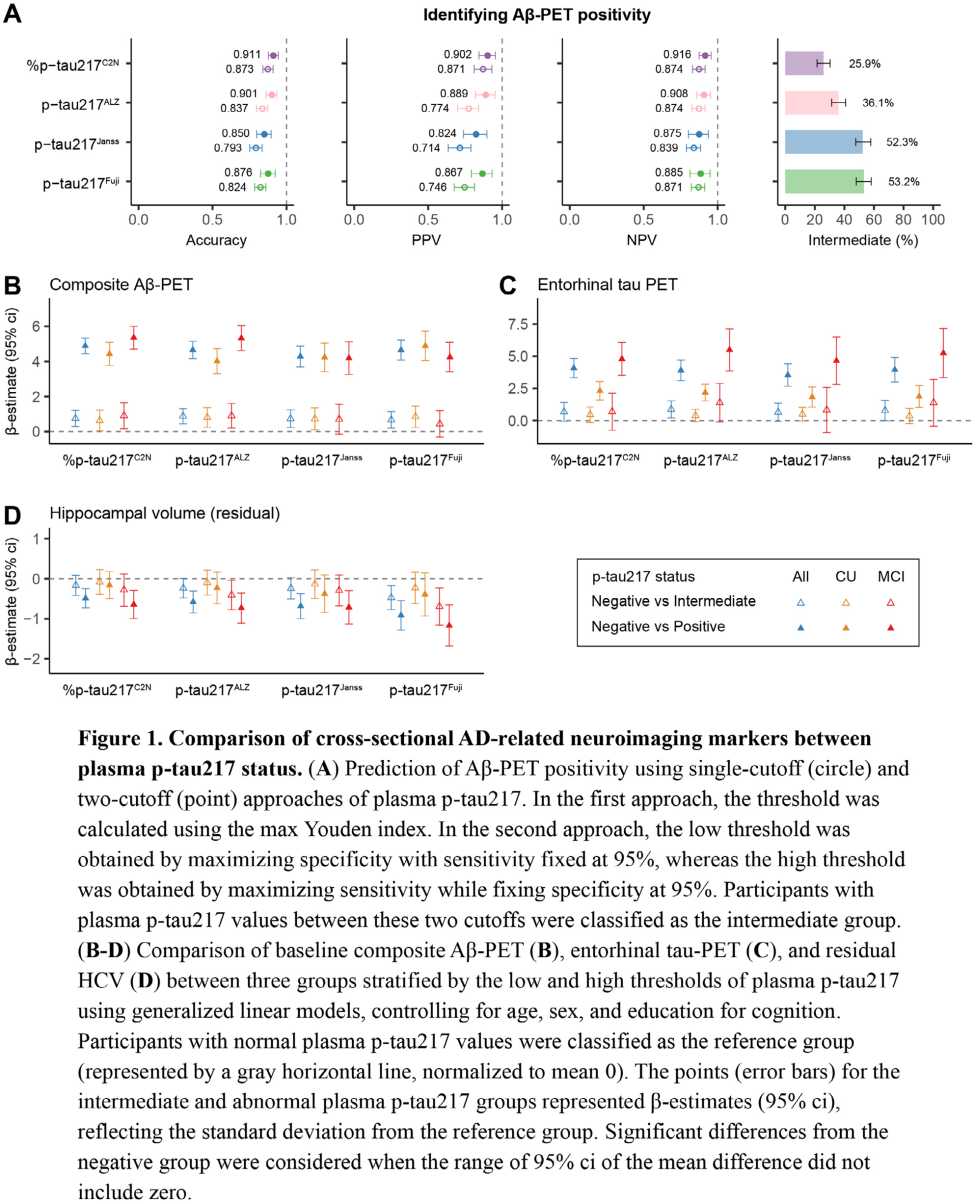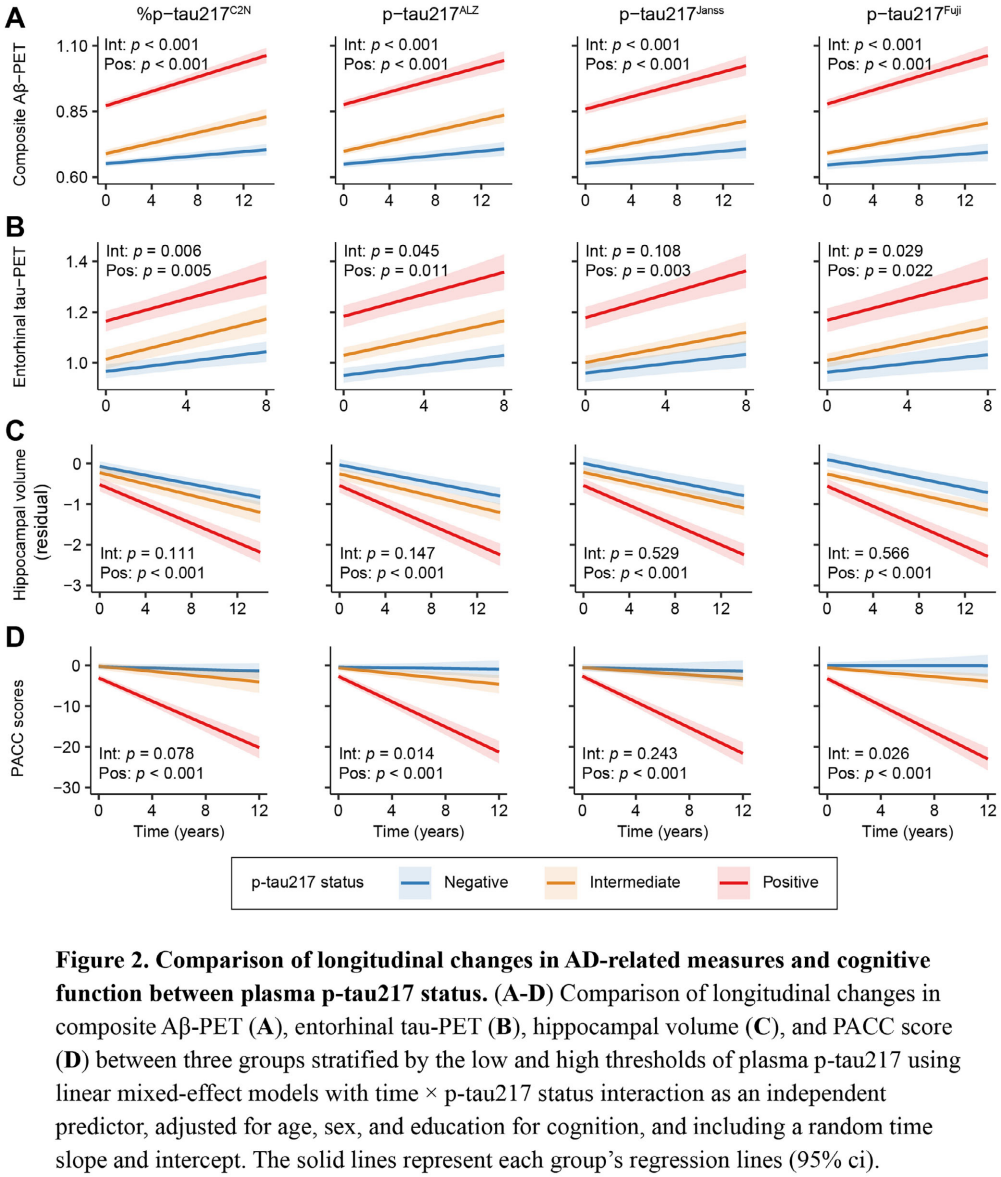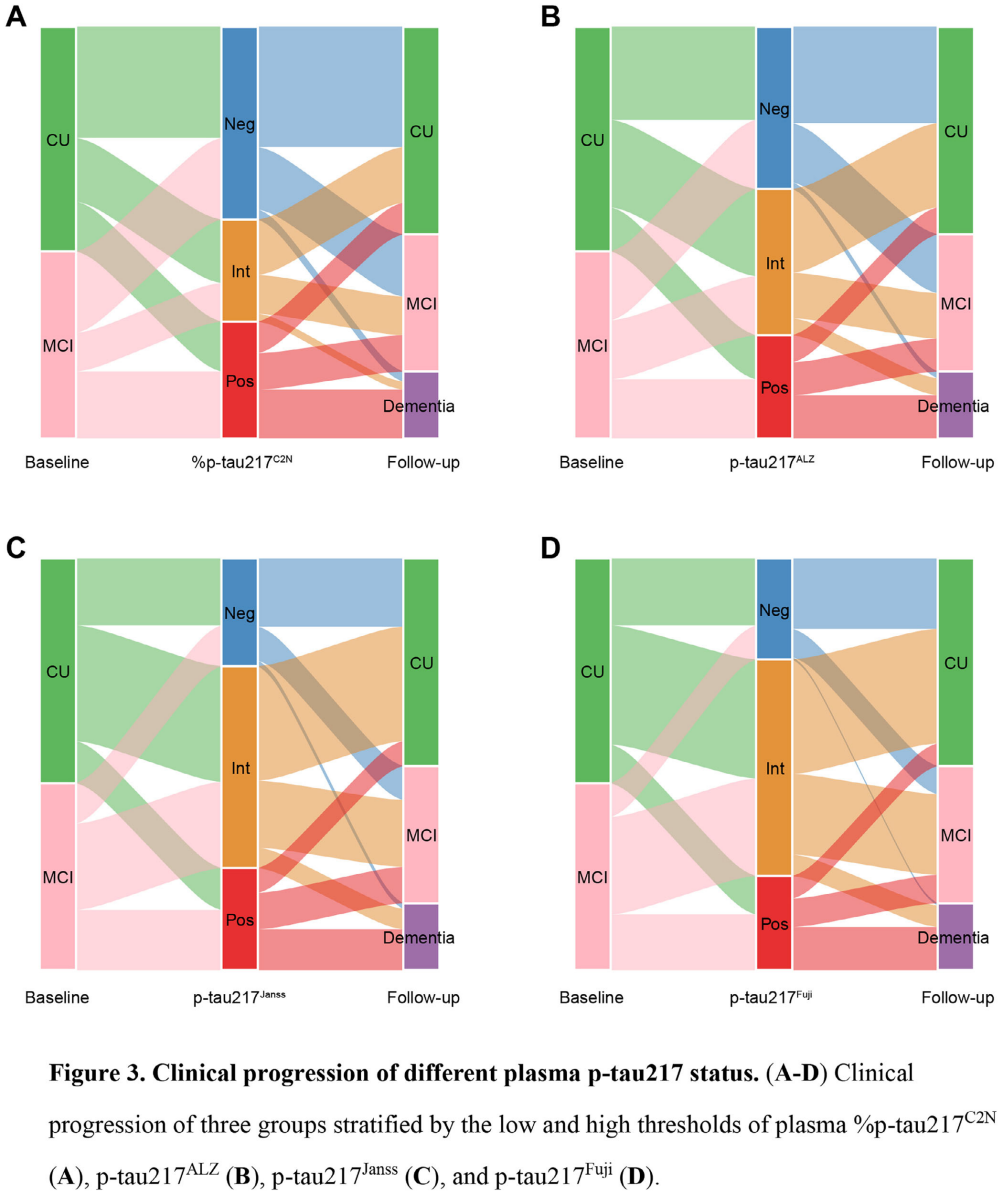# Detecting and monitoring longitudinal pathological and clinical changes of Alzheimer's disease using plasma *p*‐Tau217

**DOI:** 10.1002/alz70856_100164

**Published:** 2025-12-24

**Authors:** Guoyu Lan, Laihong Zhang, Tengfei Guo

**Affiliations:** ^1^ Institute of Neurological and Psychiatric Disorders, Shenzhen Bay Laboratory, Shenzhen, Guangdong, China; ^2^ Shenzhen Bay Laboratory, Shenzhen, Guangdong, China

## Abstract

**Background:**

Phosphorylated tau217 (*p*‐tau217) are promising blood biomarkers for evaluating Alzheimer's disease (AD) neuropathology. This study investigated the performance of leading commercial *p*‐tau217 assays in detecting and monitoring AD‐relevant processes.

**Method:**

We identified 363 non‐dementia ADNI participants with concurrent four distinct plasma *p*‐tau217 assays (C2N, ALZpath, Janssen, and Lumipulse). Participants were categorized as negative, positive, and intermediate groups according to low and high thresholds of plasma *p*‐tau217. We explored the differences in baseline and longitudinal changes in Aβ plaques, tau tangles, hippocampal atrophy, and cognitive decline across plasma *p*‐tau217 stages.

**Result:**

The two‐cutoff approach improved plasma *p*‐tau217 performance in identifying Aβ‐PET positivity, but four *p*‐tau217 assays differed in percentages of the intermediate zone (Figure 1A). Regardless of specific assays, participants with abnormal plasma *p*‐tau217 had significant abnormalities in baseline levels and longitudinal changes in Aβ‐PET, entorhinal tau‐PET, hippocampal volume, and PACC scores compared to those with normal plasma *p*‐tau217 (Figures 1‐2). Participants with intermediate plasma *p*‐tau217 also had greater baseline Aβ‐PET burden and more rapid accumulations of Aβ‐PET and entorhinal tau‐PET. Over a mean (SD) follow‐up duration of 6.8 (2.3) years, participants with abnormal plasma *p*‐tau217 exhibited higher risks of developing dementia compared to those with normal or intermediate plasma *p*‐tau217, with a larger percentage of dementia cases in the positive group (Figure 3). Notably, participants within intermediate zone also demonstrated greater risks of dementia than those with normal plasma *p*‐tau217, when using plasma *p*‐tau217^ALZ^ (Int, 11.6% vs. Neg, 4.2%), *p*‐tau217^Janss^ (Int, 10.1% vs. Neg, 5.3%), and *p*‐tau217^Fuji^ (Int, 9.9% vs. Neg, 2.2%), but not %p‐tau217^C2N^ (Int, 7.8% vs. Neg, 5.3%).

**Conclusion:**

Our findings highlight the advantages of plasma *p*‐tau217 in detecting and monitoring dynamic disease progression. Confirmatory tests and longitudinal tracking are necessary for those with intermediate plasma *p*‐tau217, as they may be at the early AD stage with high risks of rapid disease evolution. This categorized strategy may identify a novel target population for early pathological characteristics studies and interventions in AD. Incorporating high‐performance blood *p*‐tau217 tests into clinical trials may improve AD diagnosis and enable larger‐scale screening of early‐stage patients with suspected AD pathophysiology for anti‐amyloid immunotherapies.